# Current and Retrospective Childhood Ratings of Emotional Fluctuations in Adults With ADHD

**DOI:** 10.3389/fpsyg.2020.571101

**Published:** 2020-12-15

**Authors:** Astri J. Lundervold, Anne Halmøy, Emilie S. Nordby, Jan Haavik, Jocelyn I. Meza

**Affiliations:** ^1^Department of Biological and Medical Psychology, University of Bergen, Bergen, Norway; ^2^Division of Psychiatry, Haukeland University Hospital, Bergen, Norway; ^3^Department of Clinical Medicine, University of Bergen, Bergen, Norway; ^4^Department of Biomedicine, University of Bergen, Bergen, Norway; ^5^Department of Psychiatry and Biobehavioral Sciences, University of California, Los Angeles, Los Angeles, CA, United States; ^6^Department of Psychiatry and Behavioral Sciences, University of California, San Francisco, San Francisco, CA, United States

**Keywords:** ADHD, attention deficit hyperactivity disorder, emotional fluctuations, lifetime trajectories, ASRS report scale, WURS-25, MDQ

## Abstract

**Introduction:** Emotional fluctuations and mood swings are common among adults with Attention Deficit/Hyperactivity Disorder (ADHD). Here we investigated if these problems could be retrospectively traced back to childhood behavior.

**Methods:** Adults with an ADHD diagnosis (*n* = 502, 48% female) and a population-based control sample (*n* = 818, 59% female) completed the Adult ADHD Self-report Scale (ASRS), the Wender Utah Rating Scale (WURS) and the Mood Disorder Questionnaire (MDQ). All participants also provided self-reported information about co-existent psychiatric and somatic diseases, and demographic data. Reports on the MDQ were used to define subgroups with [MDQ(+)] and without [MDQ(–)] life-time periods of emotional fluctuations and the WURS scale was used to retrospectively assess childhood ADHD related behaviors and symptoms.

**Results:** 50.2% of the ADHD group and 5% of the controls were defined with emotional fluctuations [MDQ(+)]. Childhood behavior ratings of “impulsivity,” “loosing of control,” and “trouble with authorities” independently predicted emotional fluctuations reported in adulthood via logistic regression analyses. Inclusion of these three items in a classification analysis gave an accuracy score around 70% in identifying each of the two MDQ categories in the ADHD group.

**Discussion:** The strong association between self-reported features of emotional problems in childhood and in adulthood suggests a trajectory that should be detected and remediated at an early age. Future longitudinal studies should prioritize the examination of developmental mechanisms explaining the persistence of emotional problems from childhood into adulthood.

## Introduction

Attention-deficit/hyperactivity disorder (ADHD) is a childhood neurodevelopmental disorder characterized by impairing symptoms of inattention and/or hyperactivity/impulsivity that persist across situations and time (American Psychiatric Association, 2013). The prevalence of ADHD in adulthood is estimated to be 2–3% (Faraone and Biederman, 2005), and some characteristics of the disorder are associated with negative outcomes, including depressive symptomatology and self-injurious thoughts and behaviors (Hinshaw et al., 2012; Franke et al., 2018). Although not part of the core diagnostic criteria, emotional problems affect the life of many individuals with ADHD (Barkley and Fischer, 2010; Surman et al., 2013; Faraone et al., 2019), both by increasing the severity of ADHD symptoms and the presence of comorbid disorders. Co-existing emotional problems are also associated with academic and occupational difficulties with negative consequences for social and financial outcomes (Halmøy et al., 2010; Corbisiero et al., 2013; Surman et al., 2013; Shaw et al., 2014; Bunford et al., 2018). Studies have shown that 25–45% of children with ADHD have severe emotional problems (e.g., Sobanski et al., 2010; Anastopoulos et al., 2011; Banaschewski et al., 2012; Biederman et al., 2012a,b), with a prevalence increasing to 30–70% in adulthood (e.g., Barkley and Fischer, 2010; Halmøy et al., 2010; Landaas et al., 2012; Surman et al., 2013). It is important to understand these emotional problems and their trajectories from childhood to adulthood, particularly because such information may provide new opportunities for developmentally informed treatments (Aldao et al., 2010; Schweizer et al., 2020).

Although most clinicians and researchers agree on the potentially negative sequelae of emotional problems in youth with ADHD, it is still debated whether or not the emotional symptoms are part of the disorder itself (Faraone et al., 2019). Strong arguments for considering ADHD as an independent contribution to emotional symptoms and its functional consequences are given by Barkley (2015). He describes two core features giving rise to such problems in ADHD: (1) emotional impulsivity and, (2) deficient self-regulation. By this, an individual with ADHD will both have an increased reactivity to affective stimuli and problems to regulate these “high intensity” emotions back to baseline mood. In other words, emotions get out of control and leave individuals with emotions that tend to fluctuate (e.g., between happiness, irritability and sadness). Wender proposed the following triad of emotional symptoms as a characteristic for adults with ADHD; affective lability, hot temper, and emotional over-reactivity (Wender et al., 2001). Such mood swings, referred to as emotional fluctuations in the present study, are, however, not restricted to individuals with an ADHD diagnosis. Emotional fluctuations are common in the general population, particularly among adolescents (Golombek et al., 2019). Furthermore, they are associated with several other psychiatric disorders. For instance, irritability is common both among individuals with ADHD and oppositional defiant disorder (ODD; Mick et al., 2005). The feature overlap is even greater between ADHD and bipolar spectrum disorders (BSD) (Prada et al., 2014; Bunford, 2020). This overlap was documented in a study including adults with a formal diagnosis of ADHD (Halmøy et al., 2010). More than half of the adults with ADHD obtained a score above the clinical cutoff of BSD on the Mood Disorder Questionnaire (MDQ; Hirschfeld et al., 2000). This percentage was much higher than expected from self-reported lifetime history of a bipolar disorder and the proportion identified by a clinical interview based on the DSM-IV criteria. Taken together, these results support that the fluctuating emotional symptoms assessed by the MDQ are present in a large subgroup of the adults with an ADHD diagnosis.

From longitudinal studies we know that symptoms of ADHD in childhood tend to persist into adulthood (American Psychiatric Association, 2013). This includes emotional symptoms, as described by Barkley (2015). Gisbert et al. (2019) found a similar consistent trajectory from childhood to adulthood in a group of adults with ADHD reporting both current and childhood emotional symptoms. The present study followed up on these results by including items from the MDQ to define lifetime periods of emotional fluctuations and items from the Wender Utah Rating Scale (WURS; Ward et al., 1993) to assess childhood ADHD related behavior. The WURS is a self-report questionnaire based on the Utah criteria for ADHD, including items assessing emotional problems like temperament, mood instability, and emotional over-reactivity in addition to the ADHD symptoms included in the current diagnostic manual (American Psychiatric Association, 2013). Information from the WURS and the MDQ are thus both expected to identify different aspects of emotional problems, with a wider range of behavior associated with childhood ADHD covered by the WURS.

When assessing childhood symptoms and behavior retrospectively, several factors must be controlled for. Memories tend to fade over time (Miller et al., 2010), making age a relevant factor. Other studies have pointed to gender effects, where adult females tend to report more severe ADHD symptoms than males (Millenet et al., 2018; Vildalen et al., 2019). Furthermore, retrospective recall of childhood behavior is probably influenced by the high frequency of co-existing learning disabilities (Moody, 2014), somatic diseases (Instanes et al., 2018), and psychiatric disorders (Solberg et al., 2019) among adults with ADHD. Taken together, age, gender, current ADHD symptoms, and co-existent problems may all influence the recall of symptoms experienced in childhood and were therefore included as control variables in the present study.

The aim of the present study was to investigate links between emotional fluctuation in childhood (defined from WURS scores) and adulthood (defined from MDQ scores) as reported by adults with an ADHD diagnosis and by a control sample recruited from the general population. From the information referred to above we had the following hypotheses: (1) A large proportion of the ADHD group would give reports on the MDQ indicating periods of emotional fluctuations [MDQ(+)]; (2) adults defined as MDQ(+) would show greater functional impairments as evidenced by lower educational success, higher current ADHD symptoms, and more co-occurring psychiatric disorders than the ADHD group not reporting emotional fluctuations [MDQ(–)]; (3) childhood behavior reflecting emotional fluctuations will be identified as predictors of emotional fluctuations in adulthood; and (4) the predictors of emotional fluctuations in the ADHD group would also be relevant in the control group, but will probably comprise more common mood related problems than in the ADHD group.

## Methods

### Participants

The included adults were part of a larger cross-sectional study (http://www.uib. No/kgj-npd) where most of the participants in the ADHD group were recruited from a national registry of adults diagnosed in Norway from 1997 to May 2005. All participants had a formal diagnosis of ADHD before enrolled in the present study. Participants were diagnosed according to national guidelines recommended by expert teams according to the ICD-10 research criteria [World Health Organization (WHO), 2000], with allowance for the DSM-IV inattentive subtype (American Psychiatric Association, 2000), and also for comorbid disorders. Although we do not have specific information about the stage of treatment history, this should not impact the validity of the MDQ, as the MDQ assesses lifetime mood swings. The participants in the control group were randomly recruited from the Medical Birth Registry of Norway, a registry including all individuals born in Norway after January 1st, 1967 (see Halleland et al., 2012 for more information about the sample). The study was approved by the Norwegian Regional Committee for Medical and Health Research Ethics, RECWest [IRB #3 (FWA00009490, IRB00001872)]. Participants included in the present study completed the MDQ and the WURS, as well as the Adult ADHD Self Report Scale assessing current ADHD symptoms. Self-reports of childhood symptoms, dyslexia, co-existent somatic diseases and psychiatric disorders were obtained from more than 95% of the sample.

### Materials and Methods

#### Current ADHD Symptoms

Current ADHD symptoms were assessed by asking adults to complete the 18-item version of the Adult ADHD Self Report Scale (ASRS; Kessler et al., 2007). On this scale, the adults were asked to rate their ADHD-related symptoms in the past 6 months on a scale from 0 (“never”) to 4 (“very often”). In the present study we calculated the sum score across the 18 items and also created the subscales of inattention and hyperactivity/impulsivity, each including nine items. The two-factor structure and diagnostic accuracy are well-documented in previous studies (see e.g., Brevik et al., 2020).

#### Childhood ADHD Symptoms

The 25-item version of the Wender Utah Rating Scale (WURS; Ward et al., 1993) was used to retrospectively assess ADHD-related symptoms in childhood. The adults were asked to rate the presence of childhood behavior described by the items on a scale from 0 (“not at all or very slightly”) to 4 (“very much”). The items are chosen from the original 61 item version of WURS for their ability to discriminate between ADHD and controls, and the long-term reliability of WURS-25 was recently supported (see Lundervold et al., 2019). The sum score across the 25 items was used as an overall severity measure of childhood symptoms, and the search for specific predictors of emotional fluctuations in adulthood was based on information from the individual WURS items. Each item was dichotomized into those reporting that the behavior described by a given item was not at all or slightly, mildly, or moderately present (dummy coded as 0) and those reporting that the behavior was quite a bit or very much present (dummy coded as 1). WURS has demonstrated good psychometric properties, with high criterion validity (i.e., being highly predictive of an ADHD diagnosis) and its and its validity was confirmed in a recent study from our research group (Brevik et al., 2020).

#### Current Reports of Lifetime Emotional Fluctuations and Group Classifications

The Mood Disorder Questionnaire (MDQ) was originally developed to screen for Bipolar Spectrum Disorders (BSD) in clinical psychiatric patient samples (Hirschfeld et al., 2000), but has been validated for use in the general population (Hirschfeld et al., 2003), in primary care settings (Hirschfeld, 2002) as well as in samples of patients with depression (Miller et al., 2004). The first 13 MDQ items are related to lifetime periods of hypomanic/manic symptoms, answered yes/no, followed by a single yes/no question about whether the confirmed symptoms were experienced in the same time period, and then a final question evaluating the level of impairment caused by the symptoms, rated on a five-point scale (0 = not at all or slightly, 1 = mildly, 2 = moderately, 3 = quite a bit, and 4 = very much).

The ratings on the MDQ were used to classify participants into one of two mutually exclusive categories reflecting the presence or absence of hypomanic periods: (i) a screen positive category [MDQ(+)] defined according to the following criteria: seven or more answers of “yes” on the first 13 symptom items, “yes” on the next question (co-occurrence of symptoms), and at least a moderate impairment (“3 or more”) on the last item assessing impairment (Halmøy et al., 2010); (ii) a screen negative category [MDQ(–)], defined when these criteria were not fulfilled. Previous studies have shown that a positive MDQ score is correlated with a variety of psychiatric disorders (Zimmerman et al., 2011; Hardoy et al., 2015), and that it may represent a proxy for the emotional fluctuations characterizing the behavior of many adults with ADHD (Halmøy et al., 2010). For the purpose of this study, we therefore chose to use the term “emotional fluctuations” rather than hypomanic periods when describing the MDQ(+). The two MDQ categories thus represent a subgroup of the sample that currently reported a clinically defined level of lifetime emotional fluctuations, and one subgroup with such symptoms below this clinical cut-off. Severity level of emotional fluctuations is indicated by the sum score across the first thirteen items of the MDQ (MDQ-13).

#### Background Variables

The majority of the participants (>95%) reported information about gender, age, education, current occupational status, if they had been diagnosed with ADHD as a child and if they were diagnosed with dyslexia, a co-existent somatic disease (epilepsy, migraine or asthma) or other psychiatric disorders [anxiety/depression, Autism Spectrum Disorder/Tourette's, Intellectual Disability [ID], or bipolar disorder]. The lifetime presence of co-existent disorders were assessed via self-reported answers to yes/no questions (e.g., ”Do you have, or have you ever had, a bipolar disorder?”). None of the participants reported ID. A “yes” answer on the question: “Have you ever experienced significant anxiety or depression?” was used as a proxy for lifetime presence of anxiety/depression. The life-time presence of somatic disease was defined when at least one of the abovementioned diseases was reported to ever have been present.

### Statistical Analyses

Version 3.3.2 of the R statistical program (The R Foundation for Statistical Computing) was used for the statistical analyses, including descriptive exploratory analyses and group comparisons (i.e., independent samples *t*-tests for continuous variables or chi-square tests for dichotomous variables). Logistic regressions were used to identify independent predictors associated with MDQ(±) classifications. All dichotomous variables were factorized, and the dispersion parameter for binominal family was set to be one. Two preparatory steps were performed, separately for the ADHD and the control group and with the MDQ classifications as the outcome variable. First, we included potential covariates (age, gender, current ADHD symptoms, co-existing somatic and psychiatric disorders, dyslexia, and a childhood ADHD diagnosis) to eliminate covariates without significant unique contributions. Secondly, we included all the 25 dichotomized WURS variables as independent variables to select the ones with unique contributions to the MDQ- categorizations within the ADHD and the control group, respectively. The selected WURS and control variables were then included in four models, two for each group. Finally, we ran a classification analysis to investigate the prediction accuracy from the selected WURS items in the present sample.

## Results

### Descriptive Characteristics of the Sample

The control group included more females and more participants with a higher education than the ADHD group, whereas the presence of dyslexia, somatic and psychiatric disorders were more frequent in the ADHD group (see Tables 1A,B). For both groups, lifetime presence of depression/anxiety was the most frequently reported co-existing psychiatric disorder (70% of adults with ADHD). The presence of somatic diseases (i.e., epilepsy, migraine, and asthma) was significantly higher in the ADHD group than among the controls, with more than one in four with asthma also reporting migraine. Only 14% of the adults with an ADHD diagnosis reported that they obtained the diagnosis as a child (*n* = 72), of whom 74% (*n* = 53) were males. In the ADHD group, males reported higher scores than females on the MDQ-13 scale [*t*_(500)_ = 3.659, *p* < 0.001], while females reported higher scores on the total ASRS score [*t*_(500)_ = 3.557, *p* < 0.001] and the inattention [*t*_(500)_ = 3.111, *p* = 0.002] and hyperactivity/impulsivity [*t*_(500)_ = 3.351, *p* = 0.001] subscales. In the control group, males reported higher scores on the WURS scale [*t*_(816)_ = 2.347, *p* < 0.001] and a higher frequency of somatic diseases (see Tables 1A,B).

**Table 1A:** Demographic, developmental features, and clinical variables: The ADHD group, the two MDQ subgroups and the control group: Dichotomous variables.

**Frequency**	**ADHD^**∧**^** ***n* = 502**	**ADHD^**∧∧**^** **MDQ(+)** ***n* = 252**	**ADHD** **MDQ(–)** ***n* = 250**	**Controls** ***n* = 818**	**Controls**^**∧∧∧**^ **MDQ(+)** ***n* = 41**
Females (%)	48.2%^***^	41.7%^*^	54.8%	58.7%	48.8%
Education (% Uni)	22.1%^***^	15.1%^***^	29.2%	62.8%	29.3%^***^
Present work (%)	28.3%^***^	20.6%^***^	36.0%	60.6%	58.5%
Somatic diseases (%)	46.2%^***^	49.6%	42.7%	27.9%	41.5%^*^
Asthma	23.8%^***^	27.8%^*^	19.7%	11.1%	19.5%
Epilepsy	3.6%^**^	4.8%	2.4%	1.3%	2.4%
Migraine	27.4%^***^	27.1%	27.7%	18.4%	31.7%^*^
Anx/depr (%)	70.0%^***^	80.6%^***^	65.2%	13.0%	53.7%^***^
ASD/Tics disorder	10.3%^***^	10.1%	10.4%	1.1.%	4.9%^*^
Bipolar disorder	11.0%^***^	16.0%^**^	6.1%	1.0%	1.0
Dyslexia (%)	52.9%^***^	45.0%	49.2%	9.9%	39.0%^***^
ADHD as a child (%)	14.5%^***^	11.6%	17.4%	0.1%	2.4%^***^

* p < 0.05;

** p < 0.01;

*** p < 0.001.

∧ Comparison between ADHD and control group;

∧∧ Comparison between the two MDQ subgroups within the ADHD group;

∧∧∧ *Comparison between the two MDQ-subgroups in the control group*.

**Table 1B:** Demographic, developmental features, and clinical variables: The ADHD group, the two MDQ subgroups and the control group: Continuous variables.

**Mean (SD)**	**ADHD^**∧**^** ***n* = 502**	**ADHD^**∧∧**^** **MDQ(+)** ***n* = 252**	**ADHD** **MDQ(–)** ***n* = 250**	**Controls** ***n* = 818**	**Controls**^**∧∧∧**^ **MDQ(+)** ***n* = 41**
Age	33.57 (9.96)^***^	33.69 (8.80)	33.45 (11.02)	29.37 (7.43)	32.24 (5.88)^***^
ASRS	45.34 (12.59)^***^	48.85 (11.84)^***^	41.80 (12.95)	22.70 (9.61)	37.88 (13.45)^***^
Inatt	23.74 (6.66)^***^	25.25 (6.17)^***^	22.21 (6.81)	12.35 (5.14)	19.95 (7.12)^***^
Hyper	21.61 (7.15)^***^	23.61 (6.42)^***^	19.59 (7.29)	10.36 (5.46)	17.93 (7.50)^***^
MDQ-13	7.98 (3.96)^***^	10.61 (1.88)^***^	5.33 (7.73)	2.87 (3.06)	9.24 (1.77)^***^
WURS	58.32 (17.81)^***^	64.80 (16.28)^***^	51.78 (16.91)	16.88 (13.39)	40.24 (20.98)^***^

^*^p < 0.05;

^**^p < 0.01;

*** p < 0.001. ASRS, total score; Inatt, ASRS inattention subscale; Hyper, ASRS hyperactivity/impulsivity subscale; MDQ-13, total MDQ score, the first 13 items; WURS, total score.

∧ Comparison between ADHD and control group;

∧∧ Comparison between the two MDQ subgroups within the ADHD group;

∧∧∧ *Comparison between the two MDQ-subgroups in the control group*.

### The MDQ(+) and MDQ(–) Categories

In the ADHD group, about half of the adults were classified to the MDQ(+) category. Compared to those classified to the MDQ(–) category, participants in the MDQ(+) category showed lower education, reported more occupational difficulties, higher prevalence of a lifetime presence of anxiety/depression and BSD, and they obtained significantly higher scores on all the included symptom scales (see Table 1A). Within the MDQ(+) category, females with an ADHD diagnosis showed significant higher scores on the ASRS [*t*_(250)_ = 3.374, *p* = 0.004] than males, while males showed higher scores on the MDQ-13 scale [*t*_(250)_ = 2.097, *p* < 0.001].

In the control group, only 5% of the adults were classified to the MDQ(+) category, with a balanced gender distribution. The controls classified in the MDQ(+) category showed a lower education level, a higher frequency of somatic diseases, dyslexia, and lifetime presence of anxiety/depression than the controls classified to the MDQ(–) category. The differences between the two MDQ categories were statistically significant both for the ASRS [*t*_(41.771)_ = 7.522, *p* < 0.001] and its two subscales *(p* < 0.001), the WURS [*t*_(41.331)_ = 7.446, *p* < 0.001] and the MDQ-13 [*t*_(50.582)_ = 22.867, *p* < 0.001].

### Logistic Regression

#### Selection of Control Variables

Variables assumed to affect retrospective recall of childhood behavior—age, gender, current ADHD symptoms and co-existing dyslexia, somatic disease (asthma, migraine, or epilepsy), lifetime presence of anxiety/depression, ASD/tics disorders, and an ADHD diagnosis as a child—were included as independent variables with MDQ category as the outcome. Statistically significant *unique* contributions were found for both the ADHD and the control group on the lifetime presence of anxiety/depression, *p* = 0.027 and *p* = 0.001, respectively, and the score on the ASRS hyperactivity subscale, *p* < 0.001 and *p* = 0.015, respectively. In the ADHD group, a unique contribution was added for gender (*p* < 0.001), and a unique contribution from the ASRS inattention subscale was added in the control group (*p* = 0.004). The variables left with statistical significance in a given group were used as control variables in the following analyses.

#### Selection of WURS Variables With Unique Contributions

In a second preparatory step of the logistic regression analyses, dichotomized ratings on each of the 25 WURS items (not at all or slightly, mildly or moderately present vs. quite a bit or very much present) were included as predictors with MDQ category (±) as the outcome. When included together with the control variables, three WURS items showed a significant *unique* contribution to the MDQ categorization in the ADHD group: items reflecting “impulsivity,” “loosing of self-control,” and “trouble with authorities, trouble with school, visits to principal's office.” In the control group, the two items reflecting being “angry” and “not achieving up to one's potential” showed statistically significant *unique* contributions (*p* < 0.005), added by items with a weaker, but statistically significant effect at the <0.01 level, reflecting “irritability” and “trouble with authorities” and a negation of being an “overall poor student, slow learner” in childhood.

Model 1 (see Table 2A) shows the results for the selected WURS items (i.e. “impulsivity,” “loosing of self-control,” and “trouble with authorities”) in the ADHD group. All three WURS variables showed unique contributions, with ORs indicating a twofold increase in odds of being allocated to the MDQ(+) category if reporting severe symptoms on these items. The unique contributions from gender and lifetime presence of anxiety/depression reflect the higher risk in males than females and for those with anxiety/depression to be categorized as MDQ(+), added by the importance of severity level of hyperactivity/impulsivity. The unique effect from the severity level of hyperactivity/impulsivity symptoms was confirmed when adding the ASRS inattention subscale to the model: only the hyperactivity/impulsivity score was left with a statistically significant contribution.

**Table 2A:** Logistic regression models for the ADHD group with MDQ category as outcome variable.

**Model**	**B**	**B (SE)**	**Z-value**	***p***	**OR**
**Model 1**	**ADHD group**				
Intercept	−2.455	0.412	−5958	<0.001	0.086
GenderM	0.628	0.214	2.940	0.003	1.874
Anx/depr	0.657	0.223	2.943	0.003	1.929
Hyper	0.071	0.016	3.603	<0.001	1.827
Impulsive	0.710	0.254	2.797	0.005	2.033
Self-control	0.793	0.226	3.502	<0.001	2.210
Authorities	0.590	0.221	2.671	0.008	1.805

*GenderM; gender male compared to females; anx/depr; anxiety/depression where the no-category is compared with the yes category; Hyper; ASRS hyperactivity/impulsivity subscale; Impulsive; WURS item # 15; Self-control: WURS item # 18; Authorities: WURS item # 22*.

In model 2 (see Table 2B), the contributions of the WURS items identified in the ADHD group were analyzed within the control group. Although the ORs indicated that the “impulsivity,” “loosing of self-control” and “trouble with authorities” variables from WURS were important for the MDQ categorization also among controls, none of the items had a statistically significant unique contribution. As in the ADHD group, reports of lifetime presence of anxiety/depression provided a unique and strong contribution in the model. A statistically significant contribution was also found for ADHD symptoms of inattention, leaving the effect of hyperactivity/impulsivity non-significant.

**Table 2B:** Logistic regression models for the control group with MDQ category as outcome variable.

**Model**	**B**	**B (SE)**	**Z-value**	***p***	**OR**
**Model 2**	**Control group**				
Intercept	−5.416	0.725	−7.469	<0.001	0.004
Anx/depr	1.255	0.397	−3.164	0.002	3.508
Inattention	0.141	0.043	3.245	0.001	1.150
Hyper	0.069	0.040	1.726	0.084	1.107
Impulsive	0.649	0.496	1.310	0.190	1.914
Self-control	1.009	0.692	1.457	0.151	2.742
Authorities	0.924	0.505	1.829	0.067	1.520

*Anx/depr; anxiety/depression where the no-category is compared with the yes category; Hyper; ASRS hyperactivity/impulsivity subscale; Impulsive; WURS item # 15; Self-control: WURS item # 18; Authorities: WURS item # 22*.

Model 3 and 4 (Tables 2C,D) investigated the added effect of the WURS items selected for the control group. In the ADHD group (model 3), none of the added WURS items changed the unique importance of the variables reflecting “impulsivity,” “poor self-control,” and “conflict with authorities.” Although not providing unique effects, the odds were still fairly high for the items reflecting being “irritable” in childhood and feelings of not having “reached their potential” at school. In the control group (model 4), adding the variables shown to have unique contributions in the ADHD group (“impulsivity,” “loosing of self-control,” and “trouble with authorities”) increased the odds of lifetime presence of anxiety/depression, adding unique contributions from three other WURS items: being “angry” and in “conflict with authorities” as a child, and a negative effect of being an “overall poor student, slow learner.” Looking at the odds, the high OR for the item reflecting being “angry” in childhood should be noted.

**Table 2C:** Logistic regression models for the ADHD group with MDQ category as outcome variable.

**Model**	**B**	**B (SE)**	**Z-value**	***p***	**OR**
**Model 3**	**ADHD group**				
Intercept	−2.597	0.434	−5.981	<0.001	0.171
GenderM	0.646	0.217	2.982	0.003	2.928
Anx/depr	−0.654	0.225	−2.901	0.003	1.923
Hyper	0.059	0.016	3.673	<0.001	1.095
Irritable	0.111	0.226	0.419	0.675	1.878
Angry	−0.315	0.285	−1.108	0.268	1.266
Impulsive	0.704	0.256	2.746	0.006	3.359
Self-control	0.856	0.216	3.273	0.001	3.951
Authorities	0.578	0.228	2.531	0.011	2.789
Poor learner	−0.149	0.227	−0.656	0.512	1.342
Achieving potentials	0.343	0.225	1.530	0.126	2.190

*GenderM; gender male compared to females; anx/depr; anxiety/depression where the no-category is compared with the yes category; Hyper; ASRS hyperactivity/impulsivity subscale. The other variables refer to specific WURS items (see text)*.

**Table 2D:** Logistic regression models for the control group with MDQ category as outcome variable.

**Model**	**B**	**B (SE)**	**Z-value**	***p***	**OR**
**Model 4**	**Control group**				
Intercept	−5.463	0.787	−6.938	<0.001	0.004
Anx/depr	1.508	0.434	−3.476	<0.001	4.518
Inattention	0.143	0.048	3.013	0.003	1.154
Hyper	0.079	0.042	1.882	0.060	1.082
Irritable	−2.021	1.061	−1.905	0.057	0.133
Angry	2.603	1.040	2.502	0.012	13.50
Impulsive	0.858	0.519	1.653	0.098	2.359
Self–control	0.366	0.911	0.402	0.688	1.442
Authorities	0.578	0.228	2.531	0.011	2.789
Poor learner	−0.149	0.227	−0.656	0.512	1.342
Achieving potentials	0.343	0.225	1.530	0.126	2.190

*Anx/depr; anxiety/depression where the no–category is compared with the yes category; Hyper; ASRS hyperactivity/impulsivity subscale. The other variables refer to specific WURS items (see text)*.

#### Classification

Table 3 shows the confusion matrix for the ADHD group, generated by the glm procedure (caret package) in R when the WURS items reflecting childhood “impulsivity,” “loosing of self-control,” and “trouble with authorities” were included as dichotomic classifiers. When comparing the true MDQ classifications for the 502 adults in the ADHD group with the predicted classifications, 72% of the MDQ(+) cases and 66% of the MDQ(–) cases were correctly predicted by the above-mentioned WURS items. Inclusion of the control variables (anxiety/depression, ASRS hyperactivity and gender) led to an improvement of the correct classification only for the MDQ(–) category.

**Table 3:** Classification of MDQ category from childhood behavior.

**ADHD**	**WURS** * **3** **+** **control variables**		**WURS** * **3 without control variables**	
	**Actual MDQ-**	**Actual MDQ+**	**Actual MDQ-**	**Actual MDQ+**
Pred MDQ-	70% (*n* = 176)	30% (*n* = 76)	66% (*n* = 165)	28% (*n* = 71)
Pred MDQ+	30% (*n* = 74)	70% (*n* = 176)	43% (*n* = 85)	72% (*n* = 181)

WURS *3 + control variables: var + control var = “gender,” “anxiety/depression,” “hyperactivity,” “impulsivity,” “loosing of self-control,” and “trouble with authorities”.

WURS *3 without control variables: “impulsivity,” “loosing of self-control,” and “trouble with authorities”.

## Discussion

The present study showed that about half of the ADHD group and five percent of the controls were classified as having emotional fluctuations. This classification was defined from responses on the MDQ, and individuals with emotional fluctuations were allocated to what we refer to as the MDQ(+) category. Individuals in this category (from both groups) were significantly more impaired than the rest of the participants [defined as MDQ(–)]. Classification into these MDQ categories was explained by slightly different childhood symptoms in the ADHD and control groups; items reflecting childhood “impulsivity,” “loosing of self-control,” and “trouble with authorities” were unique contributors in the ADHD group, whereas being “angry, “irritable,” and having “trouble with authorities” contributed uniquely to the classification of controls. Among the control variables, lifetime presence of anxiety/depression had a significant unique contribution to explain allocation to the MDQ categories in both groups, while gender and symptoms of hyperactivity/impulsivity was added in the ADHD group and symptoms of inattention in the control group. The accuracy of predictions of MDQ category from the variables with unique contributions in the ADHD group was around 70%.

The severity of emotional fluctuations should be emphasized. Its presence was defined when an adult reported ≥ 7 impairing symptoms. A prevalence of 50% in the ADHD group corresponds to the 50.6% with features of a BSD as reported by Halmøy et al. (2010) from parts of the same sample, but is also in line with study reporting that 55% of an adult ADHD group showed extreme deficient self-regulation (Surman et al., 2013), and a study describing 60% of a group of adults with ADHD as being easily frustrated, overacting emotionally, and being easily excited/distracted by nearby activities (Barkley et al., 2008). In other words, in spite of the fact that the MDQ was originally developed to assess core symptoms of a BSD, the items seem to capture symptoms that commonly challenge functioning in a wider group of adults, including adults with ADHD. The lack of diagnostic specificity of MDQ was further supported by the much higher number of adults with ADHD classified as MDQ(+) than the number of adults reporting BSD when asked about the presence of comorbid disorders. Added to this, the 5% in the control group classified to the MDQ(+) category was also higher than expected from the single control person reporting BSD. The results from the present study thus support the diagnostic non-specificity of MDQ reported by Zimmerman et al. (2009, 2011).

There are several noteworthy results from our study. The main contribution was provided by identification of childhood behavior predicting the emotional fluctuations reported in adulthood. By this, one may speculate that some of the WURS items could be seen as a childhood version of the MDQ. The “impulsivity” and “loosing of self-control” reported from childhood overlapped well with the core features of “emotional impulsivity” and “dysfunctional self-control” described by Barkley (2015). According to Barkley, these features affect both the generation of an emotion and the ability to be self-controlled enough to react in a goal-directed manner. By this, the emotional fluctuations reported by the adults in the present study seem to be closely tied to the updating, inhibiting and shifting subcomponents of cognitive control (Miyake and Friedman, 2012). In that these components are known to be crucial for successful development of emotion regulation (see e.g., McRae and Gross, 2020; Schweizer et al., 2020), we should expect a strong effect of current symptoms of inattention on the MDQ classifications. This was only partially supported. While inattention uniquely contributed to MDQ classifications in the control group, its effect was weaker than the effect of hyperactivity/impulsivity in the ADHD group. These differential contributions are, however, probably related to the strong association between hyperactivity/impulsivity and functional impairment in individuals with ADHD (see Ahmad and Hinshaw, 2017). Symptoms of inattention may therefore still have an impact in the ADHD group, but remain secondary to the effect of hyperactivity/impulsivity symptoms when included in a logistic regression analysis.

The core childhood predictors identified by the logistic regression analyses did also show some interesting similarities and differences between the ADHD and the control group. Although the unique contribution of childhood “impulsivity” was restricted to the ADHD group, reports on the impulsivity item provided high odds for being classified to the MDQ(+) category in both groups. In the control group, this was found together with very high odds from being “angry” as a child and having “trouble with authorities.” Taken together, these results indicate that at least some adults reporting lifetime emotional fluctuations in our sample may have shown symptoms associated with behaviors frequently seen in childhood ODD. The high co-occurrence between ODD and ADHD in childhood (i.e., up to 60%; Kadesjo et al., 2003) may also explain why the controls allocated to the MDQ(+) group showed much higher severity level of ADHD symptoms than expected in a population-based sample. A more direct pathway of irritability from childhood to adulthood seems to have emerged in the ADHD group. This was supported by our finding that 80% reported “yes” to the following question on the MDQ: “Has there ever been a period of time when you were not your usual self and you were so irritable that you shouted at people or started fights or arguments?” Understanding the associations between irritability/impulsivity and negative functional outcomes is of extreme clinical importance because these links have been associated with higher risk of self-harm and suicidal ideation in both males (Conner et al., 2004) and females with ADHD (Meza et al., 2016). Future research should therefore further investigate developmental pathways from childhood impulsivity/irritability to problems associated with emotional fluctuations in adulthood, like suicidality. Understanding mechanisms of suicidality is a current research priority, given that death by suicide is the second leading cause of death among 10–34 year olds (Center for Disease Control, 2018) [CDC Web-based Injury Statistics Query and Reporting System (WISQARS), 2018].

Lifetime presence of anxiety/depression is known to have a strong impact on well-being, among adults with ADHD (see e.g., Kessler et al., 2006) as well as in the general population (see Schweizer et al., 2020). The clinical relevance of results in the present study should therefore be emphasized. First we found that the effect of anxiety/depression was not only stronger among adults with emotional fluctuations (compared to those without), but notable, more than 80% of the adults with ADHD were classified to the MDQ(+) category. Furthermore, the association between reports of anxiety/depression and the presence of emotional fluctuations was not restricted to the ADHD group. While 13% of the total control group reported lifetime presence of anxiety/depression, the percentage increased to more than 50% in the small subgroup allocated to the MDQ(+) category. This subgroup of controls was also characterized by much higher scores on current ADHD symptoms than expected in a population-based sample. These results may indicate inclusion of adults with a symptom level very close to meeting the criteria for an ADHD diagnosis, but may also reflect the significant feature overlap between symptoms of depression and ADHD (Lundervold et al., 2016). Still, our results indicate some qualitative differences between anxiety/depression reported in the ADHD and the control group. Although reported by both groups, anxiety/depression was more closely related to emotional fluctuations and childhood impulsivity in the ADHD group. The symptoms of anxiety/depression in the ADHD group may therefore be characterized within the cyclotomic and irritable domain, putting them at higher risk of emotional fluctuations in adulthood. This assumption is supported by results from previous studies, indicating that the depressive and anxious domain of temperament represents a cluster across mood disorders (Solmi et al., 2016), while the affective cyclothymic features are much more typical in adults with ADHD (Landaas et al., 2012). Future studies including more detailed information about characteristics of children and adults with symptoms of an internalizing disorder should therefore be conducted.

Although gender was not a main topic of the present study, it still warrants some comments. First, we should note that gender emerged as a unique predictor of MDQ category in the ADHD group. The effect of gender was, however, dependent on the content of the questions in the scales. As expected from previous studies, females reported higher scores on the ASRS compared to males (Vildalen et al., 2019). However, males reported higher scores on the MDQ-13 scale, indicating higher severity levels of emotional fluctuations, which provides an argument against a general reporting-bias in women. The high frequency of emotional fluctuations in both adult females and males combined with information about childhood emotional problems should also be underscored. Trajectories of these problems are likely associated with co-existing mental health problems in individuals with ADHD, including a high risk of self-injurious thoughts and behaviors. The latter is shown to be particularly high in girls with ADHD, a risk that is shown to be mediated by emotional impulsivity as well as peer rejection and victimization (Meza et al., 2016), and probably also explained by the high risk of co-existing depression, anxiety, ODD, and conduct disorder among girls (Tung et al., 2016).

The findings of this study must be interpreted in light of limitations. First, information about childhood behavior was based on retrospective recall. Although we tried to control for recall biases in the present study, classification errors show that more factors of importance should have been included in the analytic models. The lack of a more extensive diagnostic evaluation of the adults included in the study should also be listed as a limitation. As recently remarked by Faraone et al. (2019), there is a lack of standardized instruments assessing emotional symptoms in ADHD, as well as a lack of agreement upon concepts defining those symptoms. Although a more detailed analysis of the MDQ could have been used to distinguish between items more closely related to emotional problems associated with ADHD than with a BSD, we found this beyond the scope of the present study. The choice of instruments and the definition of emotional fluctuations used in the present study are limitations to keep in mind for future studies. The present ADHD sample included a very small proportion of adults who were officially diagnosed with ADHD as children. This is probably related to the recent increase in numbers of individuals being diagnosed with ADHD in adulthood and the historically low prevalence of childhood ADHD in Norway (Heiervang et al., 2007; Surén et al., 2018). The recruitment of adults with ADHD from almost all regions of Norway and controls from the population remain as strengths of the study, although the self-selection procedure may have led to a bias toward including the most well-functioning individuals with ADHD. Together with a lack of validation procedures for the classifications in the present study, characteristics of the sample may have restricted the generalizability of the results. These results should, however, serve as a springboard for future longitudinal studies of emotional problems through the lifespan of individuals with an ADHD diagnosis.

### Conclusions and Future Directions

The present study showed that emotional fluctuations reported by adults with ADHD can be traced back to similar emotional symptoms in childhood. Childhood behavior characterized by impulsivity, loosing of self-control, and trouble with authorities overlaps well with the core emotional symptoms of ADHD described by Barkley (2015). Reports of being “angry” as a child substantially increased the odds for emotional fluctuations in the control group selected from the general population. These results emphasize the importance of a comprehensive assessment following individuals with ADHD from childhood to adulthood using assessment tools including information about different aspects of emotional functioning. This information will also be important when targeting treatment options for youth to prevent future functional impairments and negative outcomes (Aldao et al., 2010; Schweizer et al., 2020). Overall, the results in the present study emphasize the importance of future longitudinal studies assessing both specific and shared characteristics across diagnostic categories within a developmental psychopathology perspective.

## Data Availability Statement

The raw data supporting the conclusions of this article will be made available by the authors, without undue reservation.

## Ethics Statement

The studies involving human participants were reviewed and approved by Norwegian Regional Committee for Medical and Health Research Ethics, RECWest [IRB #3 (FWA00009490, IRB00001872). The patients/participants provided their written informed consent to participate in this study.

## Author Contributions

AL was responsible for the design and analyses, interpretation of results, and drafting the manuscript. EN and AH commented on the drafts of the manuscripts. JH contributed as the head of the adult ADHD project and commented on drafts of the manuscript. JM commented on the design, results, and drafts of the manuscript. All authors contributed to the article and approved the submitted version.

## Conflict of Interest

The authors declare that the research was conducted in the absence of any commercial or financial relationships that could be construed as a potential conflict of interest.
